# The E670G SNP in the *PCSK9 *gene is associated with polygenic hypercholesterolemia in men but not in women

**DOI:** 10.1186/1471-2350-7-66

**Published:** 2006-07-31

**Authors:** David Evans, Frank U Beil

**Affiliations:** 1 Endokrinologie und Stoffwechsel, Medizinische Klinik III, Zentrum für Innere Medizin, Universitätsklinikum Hamburg-Eppendorf Martinistrasse 52 20246 Hamburg, Germany

## Abstract

**Background:**

Common genetic variants in the PCSK9 gene have been reported to be associated with both elevated and exceptionally low LDL levels. The association of a common haplotype, encompassing the E670G single nucleotide polymorphism, with LDL levels reported by Chen et al (J Am Coll Cardiol 2005; 45: 1644) was not confirmed by Kotowski et al (Am J Hum Genet 2006; 78:410–422).

**Methods:**

The incidence of the E670G SNP was determined in 506 patients attending the lipid clinic, University Hospital, Hamburg.

**Results:**

The frequency in men with polygenic hypercholesterolemia, 0.11 was significantly higher than in men with LDL<50^th ^percentile, 0.03, p = 0.01. In women there was no difference in the allele frequencies between the two groups.

**Conclusion:**

In a European population the E670G SNP in the PCSK9 gene is associated with increased LDL in men but not in women.

## Background

Elevated plasma LDL is recognised as a major risk factor for the development of atherosclerosis and coronary vascular disease [1]. Mutations in the *LDLR *and *APOB *genes are well established as monogenetic causes of familial hypercholesterolemia (FH) and familial defective apoB (FDB) respectively [2]. These conditions are characterised by elevated LDL and increased risk of CVD. More recently mutations in the *PSCK9 *gene [3] have been identified as the cause of a third monogenetic form of famiial hypercholesterolemia (FH3). However monogenetic hypercholesterolemia represents only a small proportion of patients with elevated LDL, in most cases hypercholesterolemia is thought to be the result of a combination of dietary and genetic factors and is refered to as polygenic hypercholesterolemia.

Since mutation in the *PCKS9 *gene can lead to monogenic hypercholesterolemia, it is possible that common genetic variation in the gene may contribute to polygenic hypercholesterolemia. Through sequencing the *PCSK9 *gene of probands with LDL below the 5^th ^and above the 95^th ^percentile, Kotowski et al [4] described a spectrum of variants which contribute to plasma levels of LDL. They found variants which were associated with low LDL and others which were associated with elevated LDL. Chen et al [5] performed a haplotype analysis of the PCSK9 gene in subjects taking part in the LCAS study. They identified a haplotype, characterised by the G allele of the 23968A>G (E670G) SNP, which was associated with increased LDL levels and confirmed their observation in probands from the TexGen study representing a normal population (mean LDL 108 mg/dl). In contrast, Kotowski et al [4] found no association of this SNP with LDL levels in subjects taking part in the Dallas Heart Study, also a population based study from Texas. The association of the IVS1-161C>T and I475V SNPs with lower LDL reported in Japenese subjects by Shioji et al. [6] could not be confirmed by Kotowski et al [4].

In this study we determine the incidence of the 23968A>G (E670G) polymorphism in the *PCSK9 *gene in patients attending the lipid outpatients clinic of the University Hospital in Hamburg. Given the conflicting reports between American and Japenese populations, we consdered it worthwhile to ascertain the role, if any, of this SNP in polygenic hypercholesterolemia in a European population. We reasoned that if this variant does indeed cause an increase in LDL then it should show a higher frequency amongst patients with polygenic hypercholesterolemia.

## Methods

### Patients

Patients were selected from those who attended the lipid outpatient clinic, Universitätsklinikum Hamburg-Eppendorf between 1997 and 2005. Informed consent was obtained and the study was approved by the local ethics commission. At the patients first visit a detailed case history was taken and biochemical and biometric values were determined. The patients had a 30–60 minute session with a dietician who discussed their normal diet and gave dietary advice. Existing therapy was, where possible, discontinued and at a second visit approximately 6 weeks later biochemical and biometric values were again determined to provide data under diet/absence of drug therapy. Only patients from whom lipid values obtained at this second visit, i.e. after the diet advice and in the absence of lipid lowering therapy were included in the study and it is this lipid value we use in our analysis. In order to eliminate patients with combined hyperlipidemia, patients with triglycerides above 200 mg/dl were excluded. A total of 506 patients were included. The clinical characteristics of the patients are presented in Table 1.

**Table 1:** Clinical characteristics of the patients

M/F	239/267
Age (years)	44 (14)
BMI (kg/m2)	25 (3.7)
Total cholesterol (mg/dl)	270 (79)
LDL (mg/dl)	194 (77)
HDL (mg/dl)	53 (17)
Triglycerides (mg/dl)	120 (40)
Lipoprotein (a) (mg/dl)	26 (32)
Hypertension (n)	98
Type 2 diabetes (n)	10
Coronary heart disease (n)	48

For continuous variables values are means (SD)

### Biochemical measurements

Plasma cholesterol (TC) and triglycerides (TG) were determined using the GPO-PAP and CHOD-PAP kits respectively from Boehringer Mannheim. HDL cholesterol was determined following precipitation of apo B containing lipoproteins with phosphotungstate (Boehringer Mannheim).

### Genotyping

DNA was extracted using QIAamp DNA Blood mini kits from Qiagen following the manufacturer's protocol. For the determination of the frequency of the 23968A>G (E670G) polymorphism a 167 bp DNA fragment was obtained following PCR with the following primers: forward primer: TACGCCGTAGACAACACG; reverse primer: TCCCCAGACACCCATCCTGG. Digestion with mnlI results in fragments of 100 bp and 67 bp for the common A allele. Carriers of the variant G allele were confirmed by digestion with sau96I.

### Statistical methods

Allele frequencies were determined by gene counting and compared using Fisher's exact test. Continuous variables were compared using the Mann Whitney test. A p value of 0.05 or below was considered statistically significant. Analysis was performed using Statistica software.

## Results

The frequency of the G allele was 0.05 (AA 458, AG 45, GG 3) which lies between that observed in the TexGen population, 0.044 (AA 291, AG 28, GG 0) and that reported for the LCAS study, 0.074 (AA 324, AG 41, GG 7) by Chen et al in their original study. There was no statistical significant difference in the frequency of the G allele in patients with LDL below the 50^th ^percentile for age and sex, 0.044 (AA 93, AG 9, GG 0), those with LDL between the 50^th ^and 95^th ^percentiles 0.064 (AA 160, AG 20, GG 1) and those with LDL above the 95^th ^percentile, 0.064 (AA 205, AG 26, GG 2). Patients with LDL above the 95^th ^percentile form a mixed group consisting of probands with monogenic hypercholesterolemia and those with polygenic hypercholesterolemia. Nearly all cases of FDB are the result of the APOB R3500Q mutation. Therefore the incidence of this mutation was determined in all the probands and eight patients were found to be carriers of the variant. Assuming that an LDL level above 300 mg/dl allows a diagnosis of familial hypercholesterolemia with 95% probability, 56 patients, 3 of whom were APOB3500 carriers fall into this group. The frequency of the G allele was 3/56 probands with LDL>300 mg/dl and 1/8 APOB 3500 heterozygotes. Removing these patients with probable monogenic hypercholesterolemia from the group of those with LDL above the 95^th ^percentile results in an increase in the frequency of the G allele in the remaining probands with polygenic hypercholesterolemia to 0.076 (AA 148, AG 22, GG 2), which although higher than that seen in patients with LDL below the 50^th ^percentile the difference is not statistically significant. Power to detect difference between polygenic hypercholesterolemia and LDL<50^th ^percentile is 0.22.

**Figure 1:**
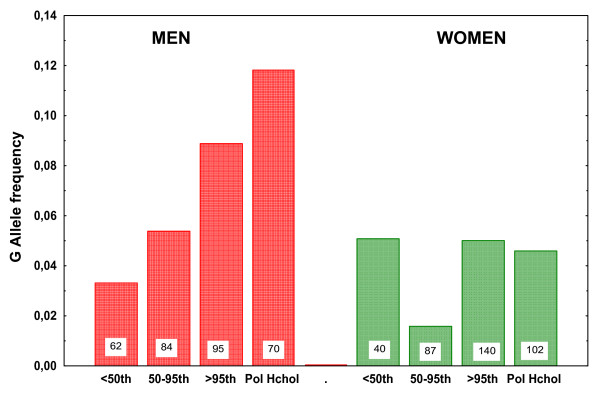
**Frequency of the G allele of the 23968A>G (E670G) SNP in the PCSK9 gene according to LDL levels**. Probands were grouped into below the 50^th ^percentile of LDL for age and sex, 50^th^–95^th ^percentile and above the 95^th ^percentile. In Pol Hchol are included probands with LDL above the 95^th ^percentile for age and sex but had neither LDL above 300 mg/dl nor were heterozygous for the R3500Q mutation in the APOB gene. The number of patients in each group is indicated.

Analysing the allele frequencies for men and women separately a different picture emerges (Fig. 1). In men the frequency of the G allele in patients with LDL below the 50^th ^percentile is significantly lower than in patients with LDL above the 95^th ^percentile, 0.03 vs. 0.09, p = 0.05. The frequency in patients with LDL between the 50^th ^and 95^th ^percentiles (0.054) was intermediate. When the probands with monogenic FH are excluded, the G allele frequency in at 0.11 higher still and is of increased statistical significance, p = 0.01. In contrast there is no association between G allele frequency and hypercholesterolemia in women.

Mean lipid values according to 23968A>G (E670G) genotype are presented in Table 2. Table 2A shows the data for all patients and in Table 2B the values when the probands with monogenic hypercholesterolemia are omitted is presented. Although there is a suggestion that the G allele is associated with increased LDL in men this was not statistically significant. In women we observed no association between E670G genotype and lipid levels. For neither sex did we observe an association between the SNP and Lp(a) as reported by Chen et al [5].

**Table 2:** Plasma lipid levels according to *PCSK9 *E670G genotype

	**MEN**			**WOMEN**	
	**EE**	**EG**	**GG**	**EE**	**EG**

**A. All patients**					

n	213	23	3	245	22
Total cholesterol (mg/dl)	251 (74)	251 (58)	279 (56)	287 (81)	285 (83)
LDL (mg/dl)	183 (74)	176 (51)	210 (70)	204 (79)	206 (86)
HDL (mg/dl)	44 (12)	47 (11)	41 (15)	60 (17)	59 (19)
Triglycerides (mg/dl)	122 (12)	136 (40)	142 (40)	117 (40)	108 (36)
Lipoprotein (a) (mg/dl)	22 (30)	20 (22)	18 (12)	29 (35)	27 (33)

**B. Monogenic Hypercholesterolemia excluded**					

n	190	23	3	210	18
Total cholesterol (mg/dl)	234 (54)	251 (58)	279 (56)	264 (56)	257 (57)
LDL (mg/dl)	166 (52)	176 (51)	210 (70)	180 (56)	174 (53)
HDL (mg/dl)	45 (12)	47 (11)	41 (15)	60 (17)	61 (20)
Triglycerides (mg/dl)	123 (40)	136 (40)	142 (40)	117 (40)	108 (20)
Lipoprotein (a) (mg/dl)	21 (30)	20 (22)	18 (12)	29 (34)	29 (23)

values are means (SD)

## Discussion

The G allele of the E670G SNP in the *PCSK9 *gene was present at an increased frequency in men, but not women, with polygenic hypercholesterolemia thus partly confirming the findings of Chen et al [5]. The majority of the probands in the LCAS study were men (310 out of 372) and in the TexGen study, two thirds of the probands were male (210 from 319), they did not separately analyse the incidence of the SNP for men and women accounting for their failure to observe a sex difference in the role of the SNP. The Dallas Study provides no information on the influence of sex on the effect of the SNP on lipid levels [4]. Whereas Chen et al [5] performed an extensive haplotype analysis we have only investigated the association with the E670G SNP which Chen et al [5] utilized as marker for the haplotype associated with LDL levels, if the haplotype structure were to be significantly different in our population this could account for differences in our findings. Since the mechanism by which variation in the *PCSK9 *gene affects LDL levels is unknown [4,5] we have no explanation for the different effect of such variation between men and women. We did not observe a significant affect of the SNP on LDL levels. This is not unexpected since we were investigating a hyperlipidemic population and since elevated LDL can be the result of common genetic variants in a number of candidate genes we would probably not detect a modest effect in this population.

## Conclusion

We present evidence that a common genetic variant in the *PCSK9 *gene contributes to polygenic hypercholesterolemia in men of European origin.

## Competing interests

The author(s) declare that they have no competing interests.

## Authors' contributions

DE carried out the molecular genetic studies, performed the statistical analysis, and wrote the manuscript. FB participated in the design of the study, was responsible for the clinical aspects and read and approved the final manuscript.

## Pre-publication history

The pre-publication history for this paper can be accessed here:


